# Assessment of the Validity and Reproducibility of a Novel Standardized Test Meal for the Study of Postprandial Triacylglycerol Concentrations

**DOI:** 10.1007/s11745-017-4275-9

**Published:** 2017-06-26

**Authors:** Nikolaos Tentolouris, Panagiotis T. Kanellos, Evangelia Siami, Elpida Athanasopoulou, Nikolaos Chaviaras, Genovefa Kolovou, Petros P. Sfikakis, Nikolaos Katsilambros

**Affiliations:** 1 First Department of Propaedeutic Internal Medicine, Medical School, Laiko General Hospital, National and Kapodistrian University of Athens, 17 Agiou Thoma Street, 11527 Athens, Greece; 2 Cardiology Department, Onassis Cardiac Surgery Center, Athens, Greece

**Keywords:** Postprandial triacylglycerols, Fat tolerance test, Precision, Agreement, Reproducibility, Repeatability

## Abstract

Lipotest^®^ is a standardized fat-rich meal designed for use as a test meal during a fat tolerance test (FTT) for the study of postprandial triacylglycerol (TAG) concentrations. Herein we examined the precision and reproducibility of examination using Lipotest^®^ on postprandial TAG levels. A total of 26 healthy consenting subjects were examined twice after 8–10 h fasting with an interval of approximately 1 week apart. Blood samples were collected at baseline and 1, 2, 3, and 4 h after consumption of the test meal for measurement of plasma total TAG levels. We examined agreement, precision, and accuracy between the two visits using the Altman plots and correlation coefficient. Reproducibility was tested using the coefficient of variation (CV) and intraclass correlation coefficient (ICC). Moreover, the area under the curve (AUC) as a summary measure of the overall postprandial TAG levels was calculated. The agreement, precision (*r* ≥ 0.74, *p* < 0.001), and accuracy (≥0.99) between the measurements in plasma TAG during Lipotest^®^ testing in the two visits were high. In terms of reproducibility, the values of CV were 15.59–23.83% while those of ICC were ≥0.75. The values of the AUCs in the visits were not different (*p* = 0.87). A single measurement of plasma TAG levels at 4 h after Lipotest^®^ consumption depicted peak postprandial TAG concentration. A FTT using Lipotest^®^ as a standardized meal has good precision and reproducibility for the study of postprandial TAG levels in healthy individuals. A single determination of plasma TAG concentration at 4 h after Lipotest^®^ consumption captures peak postprandial TAG response.

## Introduction

Cardiovascular disease (CVD) is the leading cause of death worldwide [1]. Fasting concentration of serum lipids and lipoproteins explain only in part the complex relationship between dyslipidemia and CVD [2]. Following the initial statement of Zilversmit that atherosclerosis may be a postprandial phenomenon [2], there is increasing evidence that postprandial lipemia plays an important role in the atherogenetic process because of most hours of the day are spent in the postprandial state [3, 4]. The increase in blood glucose and total triacylglycerol (TAG) concentrations following meals stimulate oxidative stress, impair endothelial function and increase inflammatory factors promoting atherosclerosis [4–6]. However, the contribution of TAG to the CVD risk remains elusive and in many studies this association was based on determination of fasting TAG concentrations [6–8].

Prospective observational studies have identified nonfasting TAG levels to be a superior predictor of CVD risk compared with fasting levels [9–14]. However, there are several methodological issues in all studies dealing with the effect of nonfasting triglyceridemia on CVD, because no standard test meal and no definite time or cut-off TAG value after meal consumption have been used to examine postprandial lipemia. In addition, postprandial TAG response depends on the amount of fat contained in the test meal and 8 h or more typically are required for a test to be performed making it cumbersome to use in a clinical setting [5]. It is apparent that standardization of a test meal and determination of the time after meal consumption for measurement of serum TAG concentrations is necessary for the study of postprandial triglyceridemia in a way similar to oral glucose tolerance test (OGTT) used for the diagnosis of diabetes and/or impaired glucose regulation [15, 16]. According to the report of the expert panel statement regarding standardized postprandial TAG testing [17], a single fat tolerance test (FTT) should be performed after an 8 h fast and should consist of 75 g of fat, 25 g of carbohydrates and 10 g of protein; a single TAG measurement 4 h after a FTT meal provides a good evaluation of the postprandial TAG response.

Lipotest^®^ (D. Genomeres Company, Athens, Greece) is a novel standardized test meal rich in fat that was developed to be used as test meal during a FTT for the study of postprandial TAG levels. In this study we examined the precision, accuracy, and reproducibility of examination using Lipotest^®^ on postprandial TAG concentrations.

## Materials and Methods

### Subjects

**Fig. 1:**
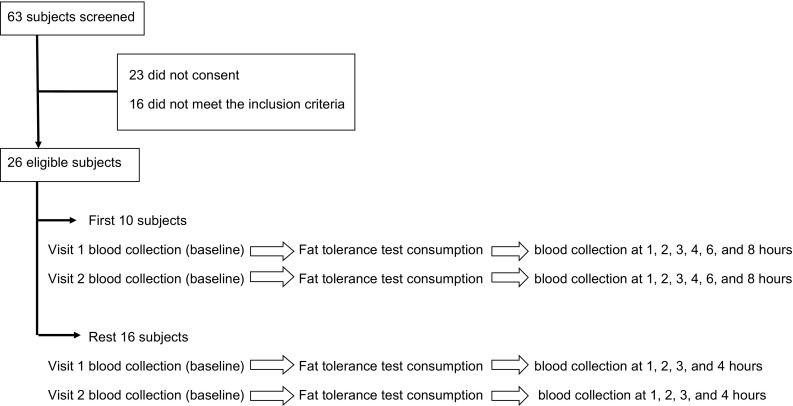
Flow chart of subject eligibility and procedure of blood collection

A total of 65 consecutive male subjects who visited the outpatient clinics of our hospital as a patient's attendant were asked to participate in the trial; of them, 23 did not consent to participate mainly because of the long duration of the test and the need to visit the clinic twice; 16 subjects did not meet the inclusion criteria and were excluded; a total of 26 subjects fulfilled the inclusion criteria and were recruited (Fig. 1). Inclusion criteria required that participants were male ≥18 years of age and had fasting TAG <220 mg/dL. Exclusion criteria were as follows: use of lipid lowering medications (statins, fibrates, bile acid resins, ω-3 fatty acids, proprotein convertase subtilisin/kexin type 9 inhibitors), alcohol or drug abuse, smoking, diabetes mellitus, history of liver, thyroid, kidney, and pancreas disease, any inflammatory condition, use of any dietary supplementation (antioxidants, vitamins/minerals, fish oil), recent history of acute illness, and treatment with medications known to affect TAG concentrations (antipsychotic, β-adrenergic blockers, protease inhibitors, interferon, raloxifene, retinoic acid drugs, sirolimus, steroids or thiazides). As the endogenous hormonal environment may impact serum TAG levels, the use of oral contraceptives and the cyclic hormonal fluctuations during the menstrual cycle may affect lipids and lipoproteins metabolism, we included only men in the study [5]. We also excluded patients with known diabetes mellitus and those found to have fasting glucose concentrations ≥126 mg/dL because defects in insulin secretion and action affect lipids and lipoproteins metabolism [5]. The subjects were instructed by a dietician to follow a weight-maintaining diet that included 50–55% of the daily energy intake as carbohydrates, 10–15% as protein, and 25–30% as fat for 3 days before each visit to the clinic. Subjects were advised to refrain from exercise and not consume caffeine or alcohol for 24 h before each visit. The demographic and clinical characteristics of the participants are shown in Table 1.

**Table 1:** Demographic and clinical characteristics of the participants

Variable	
*n*	26
Age (years), range	32 ± 1(20–64)
Height (cm)	180 ± 10
Weight (kg)	899 ± 14
Body mass index (kg/m^2^)	27 ± 4
Waist circumference (cm)	98 ± 12
Hip circumference (cm)	105 ± 8
Fasting serum glucose (mg/dL)	94 ± 22
Total cholesterol (mg/dL)	171 ± 32
High density lipoprotein cholesterol (mg/dL)	47 ± 11
Low density lipoprotein cholesterol (mg/dL)	107 ± 27
Systolic blood pressure (mmHg)	120 ± 11
Diastolic blood pressure (mmHg)	71 ± 10

Data are shown as mean ± SD. The values of fasting serum glucose, lipids, and blood pressure are the average of the two visits

The study was approved by the Ethics Committee on research on humans of the Laiko General Hospital in Athens, Greece. All participants provided written informed consent. The study protocol conforms to the ethical guidelines of the 1975 Declaration of Helsinki.

### Anthropometrics and Blood Pressure

Body weight was recorded to the nearest 0.1 kg and was measured in the morning in the fasting state with patients wearing light clothing without shoes and using a flat scale (Tanita WB-110MA, Japan). Height was measured in a stadiometer (Seca Mode 220, Hamburg Germany) and recorded to the nearest 0.1 cm. Body mass index (BMI) was calculated as weight (kg) divided by height squared (m^2^). Then, participants were asked to sit for 5 min, after which three consecutive blood pressure measurements were recorded at an interval of 1–2 min. Systolic and diastolic blood pressures were recorded by trained personnel at baseline using an OMRON HEM-907XL (OMRON, Kyoto, Japan) device.

### Blood Collection

Participants were asked to consume the Lipotest^®^ meal on two different occasions with a minimum of 3 and maximum of 7 days between each visit. Prior to each visit, subjects had fasted overnight for 8–10 h. On arrival at our laboratory, an intravenous cannula was inserted into a forearm vein, and a baseline blood sample was drawn (0 time). Then, subjects consumed the meal within 10 min; afterwards they were instructed to sit on a comfortable chair while fasted during the 4 h of the test, and only water consumption was allowed. Blood samples were collected from all participants (*n* = 26) at baseline and 1, 2, 3, and 4 h postprandially in the sitting position [18]. According to National Cholesterol Education Program Working Group on Lipoprotein Measurement, before blood withdrawal a tourniquet for <1 min was applied [18]. Moreover, we measured plasma TAG concentrations in the first ten participants in the first visit, at baseline and at 1, 2, 3, 4, 6, and 8 h after Lipotest^®^ consumption, in order to explore changes in plasma TAG concentrations (time to peak, time to decline to baseline values) during the test (Fig. 1).

### Lipotest^®^ Meal

The Lipotest^®^ meal-triglyceride tolerance test has been characterized by the National Drug Organization for Medicines of Greece as “food for specialized diagnostic triglyceride test” with Ref. No: 13664/21-02-21012,75198/23-10-12. A single serving, provided in a sachet, comprises 115 g powder that is rehydrated by adding 150 mL water. The powder and water are mixed to homogeneity (2–3 min with a hand-held mixer), and then refrigerated to form a mousse. All ingredients are food grade and are stable for a period of 24 h after preparation as proved by antioxidant tests. The composition of Lipotest^®^ ingredients is hydrogenated vegetable fat, glucose syrup solids, milk proteins, sugar, emulsifiers (lactic and acetic acid esters of monoglycerides and diglycerides), cocoa powder (20–22% fat content), defatted cocoa powder (10–12% fat content), and flavorings. The nutritional value, as well as the contribution of the Lipotest^®^ to the Daily Reference Value and European Guideline Daily Amount is depicted in Table 2. Fat used is coconut oil named Cegepal VF HC 77 and Lamequick 6068 both from Cognis, which are fully approved for use in food applications and are in powder form. The Lipotest^®^ fulfills exactly the criteria suggested by the expert panel statement regarding standardized postprandial TAG testing [17].

**Table 2:** The content and nutritional value of the Lipotest^®^ meal and its contribution to the daily required intake

Nutrient	Value	FDA DRV (%)	European GDA (%)
Energy	832 kcal	42	42
Protein	10 g	20	20
Carbohydrates	25 g	8.3	10
Sugars	14.3 g	–	16
Fat	75 g	115	109
Saturated fat	75 g	375	375
Fiber	2.1 g	8.4	
Salt	0.15 g	2.5	

*FDA* Food and Drug Administration, *DRV* daily reference value, *GDA* guideline daily amount

### Biochemistry

Venous blood samples were placed into sterile tubes containing a clot activator (Sarstedt, Nümbrecht, Germany) the biochemical measurements. Plasma was separated by centrifuging at 3000 rpm for 10 min at room temperature 10–15 min after blood collection. Fasting plasma glucose, total cholesterol and high-density lipoprotein cholesterol (HDL-C) and were measured at baseline. Triacylglycerols, TC and HDL-C were measured by an enzymatic colorimetric method using the BM Roche/Hitachi 717 analyzer (kits of Roche). Low-density lipoprotein cholesterol (LDL-C) was calculated at baseline using the Friedewald formula (LDL-C = TC − HDL-C − TAG/5). Plasma TAG concentrations were measured in fasting and postprandial blood samples. According to recommendations of the ISO 1994 and 2012 guidelines [19], all measurements were done by the same biochemistry, in the same location, using the same analyzer, the same laboratory tools, and under exactly the same conditions.

### Statistical Analysis

The Statistical Package for the Social Sciences (IBM SPSS software version 22.0 for Windows, Armonk, NY, USA) and the Medcalc Software (version 12.2.1.0, Medcalc, Ostend, Belgium) were used for the analyses. Data were tested for normal distribution of the values using the Kolmogorov–Smirnov test. The values of the normally distributed data are shown as mean ± SD. Because TAG levels were not normally distributed, the geometric mean (95% confidence intervals), as derived by log transformation were calculated and used in the analyses. The paired samples *t* test (for parametric data) or the Wilcoxon test (for nonparametric data) was used to compare differences between the two visits. Agreement between plasma TAG concentrations at different time-points was performed by Bland–Altman plots [20]. The Pearson correlation, an index of the precision, was applied to evaluate the correlation coefficient of plasma TAG values between the two visits. The concordance correlation coefficient (CCC) was calculated and evaluated as proposed by Lin [21]. The CCC evaluates both the precision and accuracy of the relationship between two measurements, and is the product of the correlation coefficient (*r*) between paired measures and a bias correction factor (*C*_b_) that measures how far the best-fit line between them deviates from the 45° line; the values range from 0.00 (no agreement) to 1.00 (perfect agreement). *C*_b_ is a measure of the accuracy [21]. Reproducibility of postprandial lipemia after Lipotest^®^ consumption was assessed by coefficient of variation (CV = 100 × SD/mean) and intraclass correlation coefficient (ICC). The ICC values ≥0.75 are interpreted as excellent reproducibility [22]. In addition, the area under the curve (AUC) was used as a summary measure to examine the overall postprandial response in plasma TAG levels during the experiment in both visits using the trapezoid rule. Maximal changes (iC_max_) were calculated by subtracting baseline concentrations from the maximal ones. Differences in iC_max_ were analyzed using univariate analysis of variance and time to reach maximal concentrations (*T*_max_) were analyzed using a nonparametric Sign test. The power of the study was estimated after the experiment; a total of 25 participants offered a power of >0.80 at *a* = 0.05 for the detection a mean difference in AUC of 5 mg × *h*/dL between the two visits. Significance (two-tailed) was set at *p* < 0.05.

## Results

**Fig. 2:**
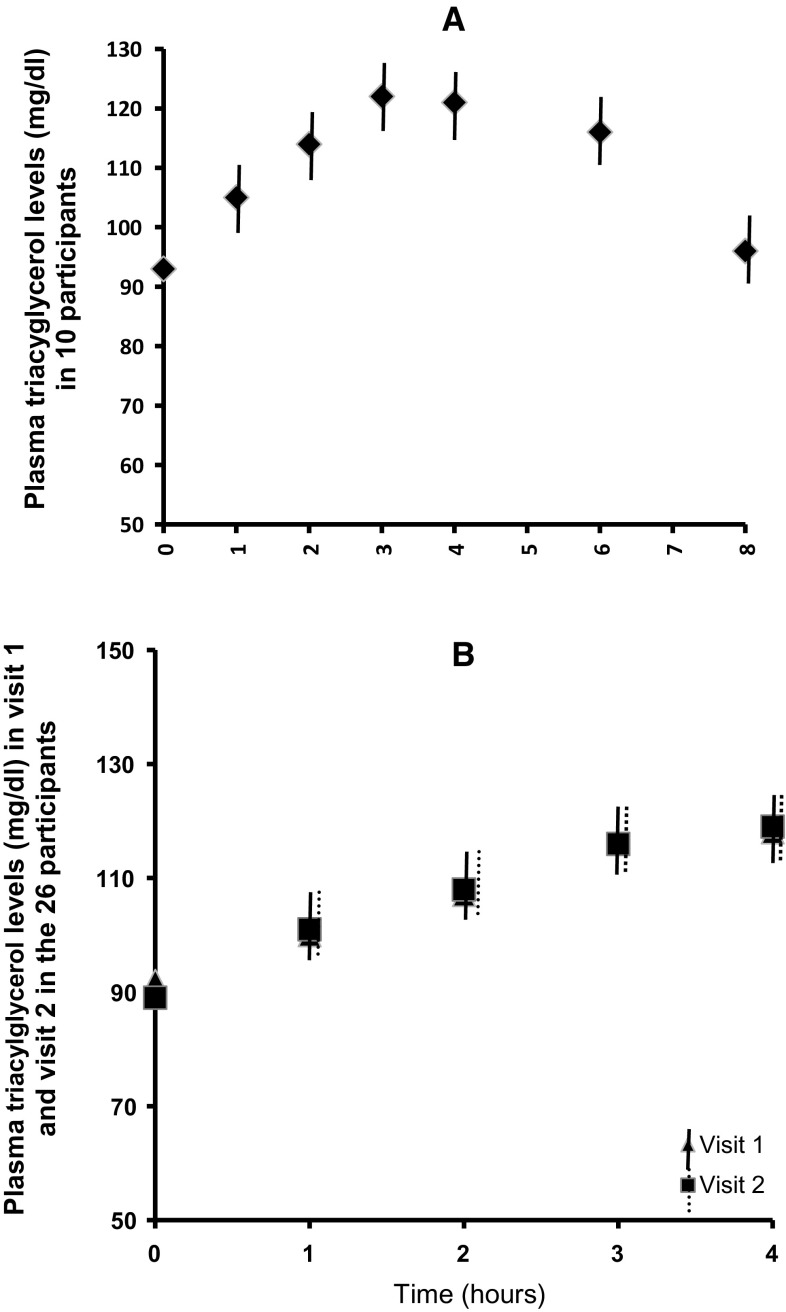
Plasma triacylglycerol levels measured once in the first 10 participants up to 8 h after Lipotest^®^ consumption (**a**) and plasma triacylglycerol levels in the 26 participants up to 4 h after Lipotest^®^ consumption (**b**). Data are shown as mean ± standard error (*vertical lines*) of mean

Participants were on average overweight, and the age range was 20–64 years (Table 1). At the beginning of the study we measured plasma TAG concentrations in ten participants for 8 h after Lipotest^®^ consumption. We found that in all subjects, plasma TAG levels peaked until the 4th hour; afterwards, in four subjects they remained approximately stable until the 6th hour and then they declined, while in six subjects plasma TAG concentrations declined after the 4th hour. In all participants plasma TAG concentrations returned to baseline levels by 8 h postprandially (Fig. 2a). Therefore, we considered that the 4-h period after consumption of the Lipotest^®^ was sufficient for the study of postprandial lipemia and analyses were performed for this duration of the study.

**Fig. 3:**
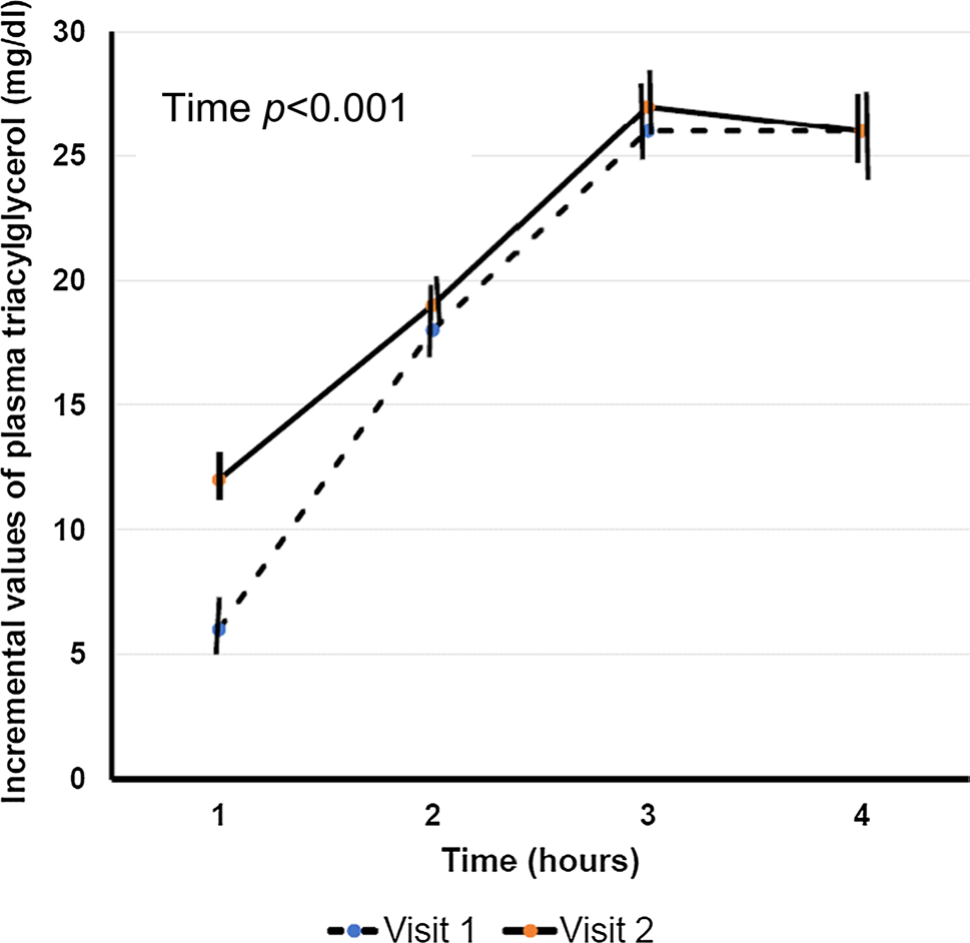
Changes in plasma triacylglycerol concentrations in visit 1 (*continuous line*) and visit 2 (*dashed line*). Data are presented as mean ± standard error (*vertical lines*) of mean (*n* = 26). Changes from baseline values were analyzed using linear mixed models with visit and time as within-subject fixed factors and subject identification number as between-factor

In the 26 participants, after Lipotest^®^ consumption there was an average increase in plasma TAG concentrations. The geometric mean iC_max_ of plasma TAG was 21 mg/dL and 24 mg/dL and the iT_max_ 170 min and 162 min in visit 1 and visit 2, respectively, with no statistically significant difference (*p* > 0.05) between the two visits (Fig. 3; Table 3). Figure 3 shows the change in postprandial TAG concentrations, which gradually increased and peaked 3 h after Lipotest^®^ consumption (*p* value for time <0.001). The postprandial pattern was comparable in the two visits after Lipotest^®^ intake (*p* value visit × time = 0.15). The individual postprandial response varied and in two subjects with fasting plasma TAG ≤80 mg/dL there was no increase in plasma TAG. Moreover, the peak value in most of the participants was observed at 3 h and in two subjects at 4 h (Figs. 2b, 3).

**Table 3:** The values of serum triacylglycerol levels (mg/dL) in visit 1 and visit 2

	Visit 1	Visit 2	*p* value	*r* value	CCC-value	Accuracy	CV%
Baseline (0 h)							
Arithmetic mean ± SD	92 ± 44	90 ± 46					15.59
Geometric mean (95% CI)	81 (60–100)	79 (64–97)	0.54	0.85	0.85	0.99	4.42
1 h							
Arithmetic mean ± SD	100 ± 45	101 ± 51					23.83
Geometric mean (95% CI)	90 (74–110)	90 (74–110)	0.97	0.74	0.74	1.00	5.35
2 h							
Arithmetic mean ± SD	107 ± 54	109 ± 58					18.75
Geometric mean (95% CI)	93 (74–118)	96 (78–118)	0.40	0.84	0.83	0.99	4.81
3 h							
Arithmetic mean ± SD	116 ± 61	116 ± 66					14.92
Geometric mean (95% CI)	102 (82–126)	100 (79–126)	0.66	0.90	0.89	0.99	3.72
4 h							
Arithmetic mean ± SD	118 ± 60	115 ± 65					17.21
Geometric mean (95% CI)	104 (84–129)	99 (79–124)	0.30	0.88	0.87	0.99	4.10
iC_max_ (mg/dl)							
Arithmetic mean ± SD	30 ± 26	33 ± 27					
Geometric mean (95% CI)	21 (12–33)	24 (16–37)	0.65				
*T*_max_ (min)							
Arithmetic mean ± SD	180 ± 54	156 ± 54					
Geometric mean (95% CI)	170 (150–196)	162 (122–175)	0.17				

*p* values are the comparison of the geometric means between visit 1 and visit 2

*SD* standard deviation, *CI* confidence intervals, *r* Pearson correlation coefficient (all values are statistically significant with *p* < 0.001), *CCC* concordance correlation coefficient, *CV* coefficient of variation, *iC*_*max*_ maximal triacylglycerol concentrations, *T*_max_ time to maximal triacylglycerol concentrations

**Fig. 4:**
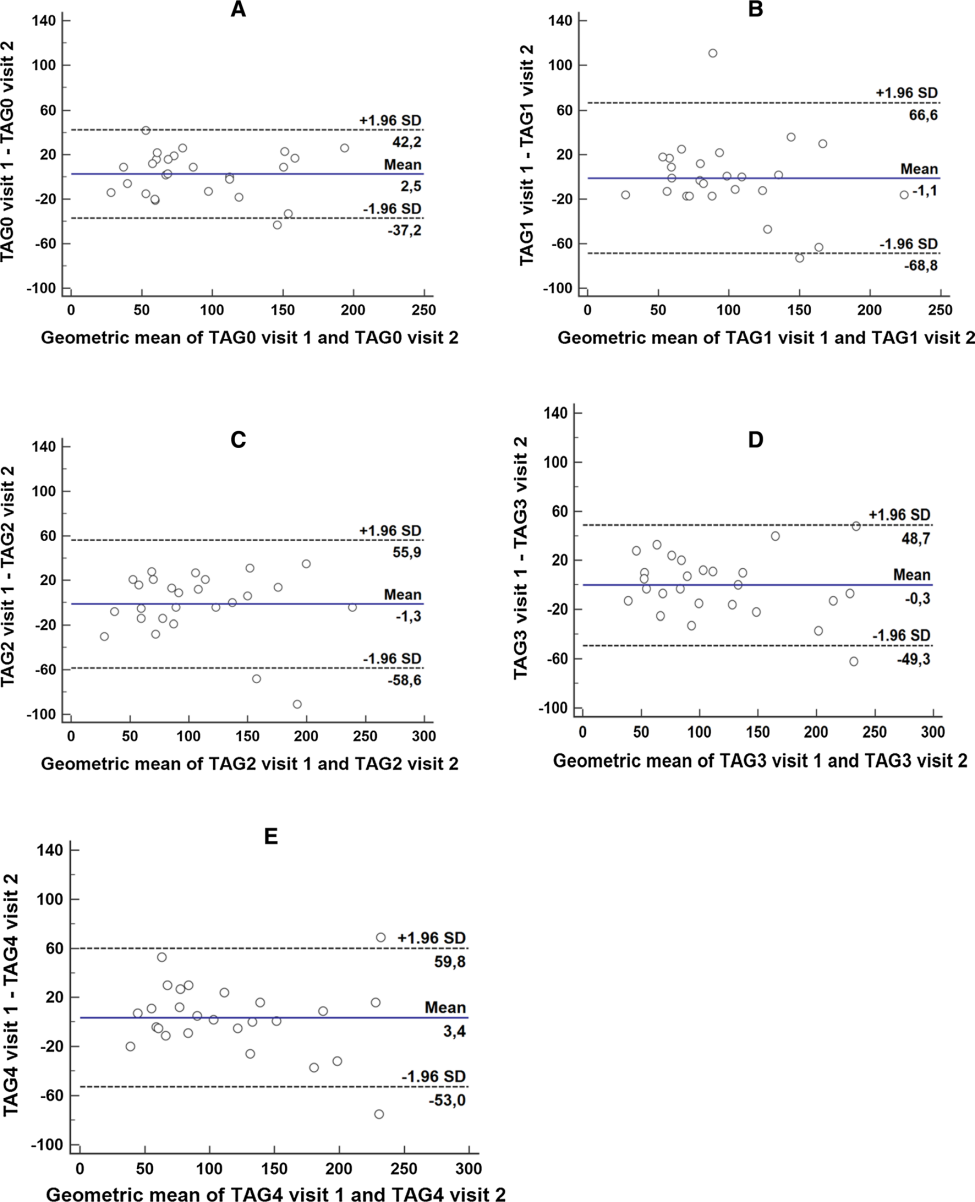
The Bland–Altman plot shows the agreement of the plasma triacylglycerol measurements at baseline (0 h) (**a**), at 1 h (**b**), at 2 h (**c**), at 3 h (**d**), and at 4 h (**e**). The *middle continuous dark line* depicts mean difference and the *outer dark dashed lines* show the upper and lower 95% limits of agreement. *TAG0 visit 1* the baseline value of plasma triacylglycerol levels at visit 1, *TAG0 visit 2* the baseline value of plasma triacylglycerol levels at visit 2, *TAG1 visit 1* the value of plasma triacylglycerol levels at 1 h at visit 1, *TAG1 visit 2* the value of plasma triacylglycerol levels at 1 h at visit 2, *TAG2 visit 1* the value of plasma triacylglycerol levels at 2 h at visit 1, *TAG2 visit 2* the value of plasma triacylglycerol levels at 2 h at visit 2, *TAG3 visit 1* the value of plasma triacylglycerol levels at 3 h at visit 1, *TAG3 visit 2* the value of plasma triacylglycerol levels at 3 h at visit 2, *TAG4 visit 1* the value of plasma triacylglycerol levels at 4 h at visit 1, *TAG4 visit 2* the value of plasma triacylglycerol levels at 4 h at visit 2

The agreement of plasma TAG concentrations between the two visits was high as shown by Bland–Altman plots (Fig. 4a–e). Pearson correlation coefficient was high and it was highest for the values at 3 and 4 h (all *p* < 0.001). The accuracy of the results was high (≥0.99 at all time-points) (Table 3). The CV of the arithmetic data were 16, 24, 19, 15, and 17% for the baseline values, the values at 1, 2 3, and 4 h, respectively, of the study, and it was <6% for the log-transformed values. The CCC was 0.85 for the baseline values; it was 0.75 at 1 h, 0.83 at 2 h, 0.89 at 3 h, and 0.87 at 4 h (Table 3). The ICC values were ≥0.75 at all time-points. The mean differences of plasma TAG between the measurements in the two visits were low. The confidence limits of the ICCs and the limits of agreement for plasma TAG were narrow (Table 4). The geometrics means of the AUCs in two visits were similar and ratio of the geometric means was not statistically significant (*p* = 0.87) (Table 5).

**Table 4:** Agreement of serum triacylglycerol measurements between visit 1 and visit 2

	Difference (mean ± SD)	95% limits of agreement		Confidence limits		
		Lower	Upper	ICC	Lower	Upper
Triacylglycerols at baseline	−0.014 ± 0.120	−0.063	0.034	0.860	0.751	0.935
Triacylglycerols at 1 h	−0.009 ± 0.15	−0.0062	0.060	0.756	0.524	0.883
Triacylglycerols at 2 h	0.010 ± 0.136	−0.044	0.066	0.841	0.677	0.925
Triacylglycerols at 3 h	−0.009 ± 0.106	−0.052	0.033	0.902	0.902	0.955
Triacylglycerols at 4 h	−0.024 ± 0.116	−0.071	0.023	0.879	0.751	0.943

The difference is between the geometric means of serum triacylglycerol values at visit 1 and visit 2

*ICC* intraclass coefficient of variation

**Table 5:** Comparison of the area under the curve of serum triacylglycerol values at visit 1 and visit 2

	Area under the curve
Visit 1	
Arithmetic mean ± SD	429 ± 204
Geometric mean (95% confidence intervals)	387 (316–475)
Visit 2	
Arithmetic mean ± SD	428 ± 226
Geometric mean (95% confidence intervals)	392 (317–484)
Statistics on log-transformed scale	
Difference	−0.009
95% confidence intervals of difference	−0.130 to 0.112
Test *t* statistic	−0.153
Degrees of freedom	50
*p* value (two-tailed)	0.87
Back-transformed results	
Ratio of geometric means	0.978
95% confidence intervals of ratio	0.740–1.294

**Fig. 5:**
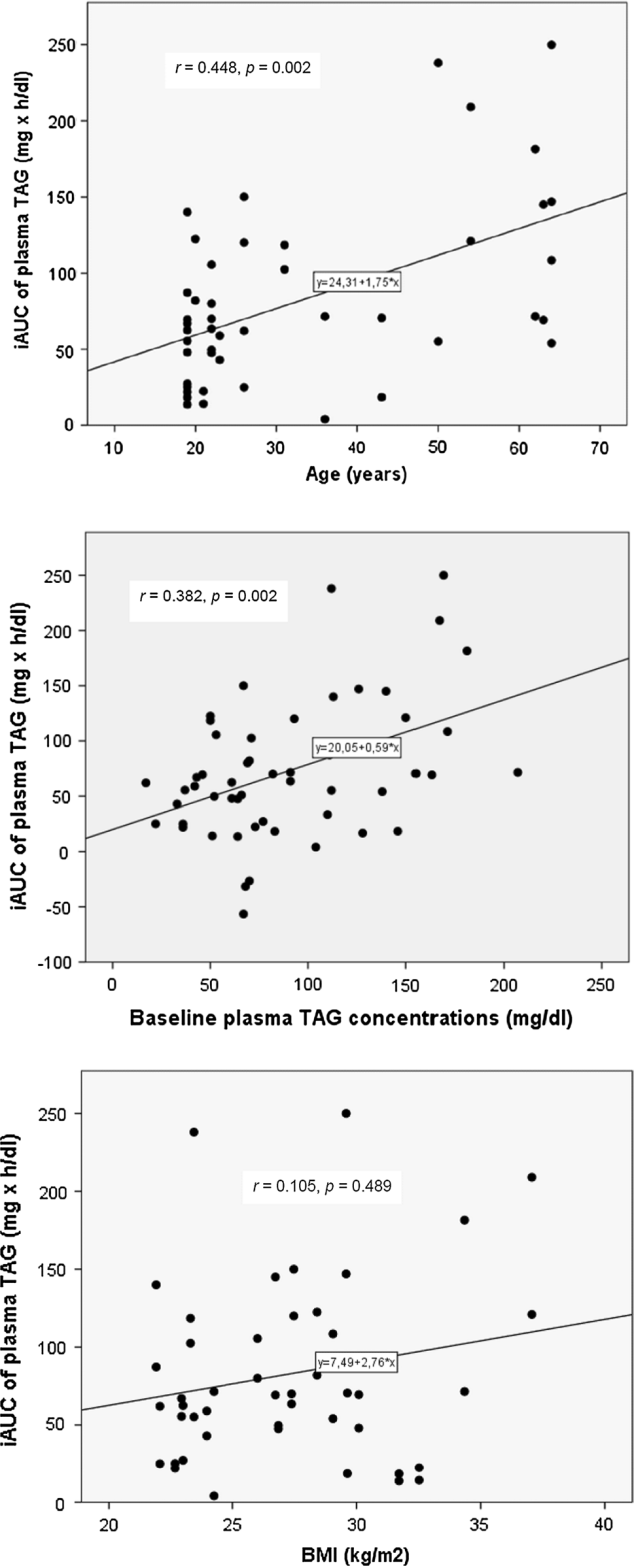
Correlations between incremental area under the curve (iAUC) of plasma triacylglycerol (TAG) concentrations and age, fasting TAG concentrations, and body mass index (BMI)

The geometric mean (95% CI) of the incremental AUC (iAUC) of plasma TAG was not significantly different between the two visits [45 (27–75) mg × *h*/dL in visit 1 and 48 (29-81) mg × *h*/dL in visit 2, *p* = 0.83). iAUC correlated significantly with age (*r* = 0.448, *p* = 0.002) and baseline plasma TAG levels (*r* = 0.382, *p* = 0.002); no significant correlations were found with BMI (*r* = 0.105, *p* = 0.489) or waist circumference (*r* = 0.178, *p* = 0.236) (Fig. 5).

Consumption of Lipotest^®^ was well tolerated by 25 of the participants; one of them felt fullness 160 min after consumption of the meal; no other adverse events were observed.

## Discussion

The main finding of this study is that using Lipotest^®^ as a standard test meal it is possible to study postprandial TAG response with high precision and good reproducibility in healthy male individuals. In addition, it was shown that a single determination of plasma TAG concentration at 4 h after Lipotest^®^ consumption is adequate to capture the peak postprandial TAG response.

The results of large-scale trials have confirmed the importance of non-fasting TAG levels in the pathogenesis of atherosclerosis [9–14]. However, reliable determination of non-fasting TAG levels has remained an important unresolved issue and TAG have traditionally been measured in the fasting state for three main reasons [17, 23]; firstly, postprandial values of blood TAG have large variability in comparison with the fasting values; secondly, fasting TAG is a necessary component for the Friedewald equation in order to estimate LDL-C [23]; and thirdly, no standard test was available nor the time after meal consumption for determination of postprandial TAG was known [17, 24].

In this study, we used the Lipotest^®^ as a standard test meal to examine postprandial TAG concentrations. Lipotest^®^ contains 832 kcal and its main content is saturated fat (75 g), but contains also 25 g carbohydrates and 10 g protein. It is estimated that a total of 70–80 g of fat is ingested normally during a whole day by many humans, since most meals contain 20–40 g of fat with usual daily pattern of up to four meals [25]. Mihas *et al.*, in a meta-analysis evaluated 113 trials on postprandial TAG concentrations in healthy subjects; they concluded that the ideal amount of fat in the test meal that led to the highest standardized mean difference in TAG values at both 4 and 6 h, compared with fasting values, should preferably be 70–80 g [26]. A limitation of using an unusually high amount of fat in a FTT meal could be that subtle differences might be overshadowed by an exaggerated postprandial TAG accumulation due to the excess fat load [17]. This should be further evaluated through dedicated studies. Because absorption of fat depends partly on presence of some carbohydrates and protein in the meal to ensure the full range of metabolic responses involved in the postprandial TAG variations [27, 28], it has been suggested that a FTT meal should be a mixed meal consisting of 75 g fat, 25 g carbohydrates and 10 g protein [17]. Lipotest^®^ is a meal with content proper for a FTT, fulfilling the criteria proposed by literature data and the expert panel for clinical purpose [17].

The fat content in Lipotest^®^ is emulsified fat. It is known that the type of fat (spread or emulsified) may have impact on postprandial TAG response, because emulsified fat is absorbed faster, peaks earlier, and postprandial lipemia is more pronounced in comparison to the spread fat [29]. Vors *et al.* reported that obese men presented a delayed increase of chylomicron-TAG after consumption of the spread fat; interestingly, no difference was found between normal-weight and obese individuals after consumption of emulsified fat [29]. We also found that the iAUC after Lipotest^®^ consumption was independent of the BMI or waist circumference. Therefore, Lipotest^®^ is very useful to diagnose impaired postprandial lipemia independently of the BMI or other subtle metabolic differences.

Moreover, it is recognized that all fats are not equal in metabolic impact despite equal energy content. Thus, lipid structures, evaluated at scales ranging from the molecular to the supramolecular ones, lactic and acetic acid esters of mono- and diacyl-glycerol can impact metabolism, including their interactions with the food matrix, impacts fatty acids intestinal absorption and post-absorptive metabolism [30]. In addition, hydrogenated vegetable fat can impact inflammation [31, 32] or gut microbiotica inducing colitis and metabolic syndrome [33]. These possible nutritional impacts should be considered in the development of new food formulations with enhanced taste and texture for the long-term consumption; however, we do not expect a detrimental effect after consumption of a single meal in the form of a FFT.

Plasma TAG is a non-discriminatory marker of all TAG carrying lipoproteins of both intestinal and hepatic origin, which are distributed over a wide range of size and density. Therefore, a subject of debate in the literature is what to measure during a FTT, since in many studies plasma total TAG concentrations were measured [12–14], whereas others measured chylomicrons (CM) [34, 35], CM-TAG [36, 37], very low-density lipoproteins VLDL [39], and intermediate density lipoproteins [36, 38] or apolipoprotein AIV [39]. Since fasting and non-fasting TAG are acceptable as risk factors for CVD events and its determination can be easily performed, it has been suggested to measure total TAG for the evaluation of postprandial lipemia after a standardized FTT [17].

With regards to the time of blood sampling after a FTT, the data from a meta-analysis of 113 clinical trials in healthy subjects [26] showed that measuring TAG concentration at the 4 and 6 h after a FTT is representative of postprandial TAG response. Previous also studies that have investigated postprandial TAG have showed that 4 h after a FTT is the most representative time to measure the TAG response [12, 40]. Weiss *et al*. examined postprandial response of plasma TAG on four different occasions before and every hour for 8 h after consumption of a fat-rich meal with a caloric and fat content like that of Lipotest^®^. They found that measurement of plasma TAG at 4 h is a valid time for determination of postprandial TAG [40]. The same authors described that the TAG responses from the 4-h test accounted for 89–96% of the variance in the 8-h test results [40]. In the Women’s Health Study, TAG measured 2–4 h postprandially had the strongest association with CVD events [13]. Also, the Copenhagen General Population Study reported peak TAG levels at approximately 4 h after a FTT, as well as after normal meals [12, 41]. We also found that in most of the participants with fasting TAG levels >80 mg/dL, the peak value of plasma TAG was observed at 3–4 h after Lipotest^®^ consumption. Therefore, literature data and the findings of the present study agree that if we want a single measurement to capture postprandial TAG response, the best time is 4 h after consumption of either Lipotest^®^ or a fat-rich meal. A single measurement of plasma TAG in the postprandial state has the advantage of simplicity and makes the Lipotest^®^ or a FTT as proposed by the expert panel statement and discussed above [17] easy for implementation in both research and clinical practice.

Triacylglycerols have long been the most problematic lipid measure in analyses because the distribution is markedly skewed, which necessitates categorical definitions or log transformations and the variability is high and increases with the level of TAG [4]. In this study, we showed that both precision and accuracy of determination of plasma TAG during the two visits of the study was high. The agreement between the values of plasma TAG during the two visits assessed by Bland–Altman plots was excellent. However, the range of CCC values was 0.74–0.89 that is considered suboptimal. In terms of reproducibility, the CV value at 4 h was 17.21% for the normal and 4.10% for the log-transformed values. Commonly, a CV value of <15% as acceptable for bioanalytical assays [42]. Previous data demonstrated that the ICC value for fasting TAG was above the 0.75 cut point for high reproducibility and the same was valid for the AUCs for TAG derived from the abbreviated 4-h test [40]. Our findings agree with these data and further support those of Brown *et al.* who reported that postprandial TAG concentrations measured 3.5 and 9 h after a high-fat meal were highly reproducible (ICCs = 0.76 and 0.85, respectively) [43]. Gill *et al.* examined postprandial TAG response in men and women of reproductive age after a fat tolerance test performed twice with an interval of 1 week [44]. They found that there was no significant difference in postprandial TAG responses between the two visits in men; the intraclass correlation coefficient between the two visits was 0.93, and the within-subject coefficient of variation was 10.1%. However, in women, the postprandial TAG response was lower in the luteal phase than in the follicular phase; the intraclass correlation was 0.65 and within-subject coefficient of variation was 23.2%. These results suggest that, with adequate control of preceding lifestyle, reproducibility of postprandial TAG responses is high in men, but menstrual phase should be taken into consideration when studying these responses in women of reproductive age [44].

Another test that is used widely for the diagnosis of diabetes and/or impaired glucose regulation is the OGTT [15]. Although low reproducibility of OGTT is a key shortcoming of this assay [45–48] and the 2 h CV of blood glucose is 25% [45], it is considered as one of the standard methods for the diagnosis of impaired glucose regulation and/or diabetes, because it was recognized that fasting hyperglycemia was a too late criterion for the early diagnosis of diabetes [15]. However, since blood glucose levels, as well as the level of most metabolites in blood including TAG can widely vary, and methods or ways for minimizing the normal fluctuations of biological phenomena are not possible, it is necessary to standardize the conditions before and during the test in order to minimize variability of measuring blood TAG [18]. In addition, it is known that the dinner composition before the day of the examination can impact post-meal lipemia [49–51]. In our study, no specific recommendation was given for the dinner composition before the visit to the clinic, but prior to each visit, subjects had fasted overnight for 8–10 h. Despite no specific standardization of the dinner before examination was asked by the participants, under the condition that control of preceding lifestyle was adequate, this did not impact repeatability.

Based on studies that have evaluated postprandial TAG [9, 10, 12–14, 26], consensus statements on the topic [17], and the recent recommendations by the Joint Consensus Statement from the European Atherosclerosis Society and European Federation of Clinical Chemistry and Laboratory Medicine [52], fasting TAG ≥150 mg/dL and non-fasting TAG ≥175–180 mg/dL are considered abnormal. Overall, 31% of the adult US population has a fasting TAG level >150 mg/dL with no appreciable change between NHANES 1988–1994 and 1999–2008 [53] and the data based on roughly 25,000 men and 25,000 women from the Copenhagen General Population Study suggest that 38% of men and 20% of women have undesirable non-fasting TAG levels >180 mg/dL [41]. It is suggested that subjects at high risk for CVD with blood TAG levels between 89 and 180 mg/dL could possibly be benefited diagnostically by being tested postprandially with Lipotest^®^. Individuals who have fasting TAG concentrations <89 mg/dL commonly do not have exaggerated or delayed response of TAG to a FTT and will not benefit diagnostically from a FTT [17]. On the other hand, individuals with fasting TAG >180 mg/dL usually have exaggerated and delayed response of TAG to a FTT, and therefore, will not benefit diagnostically from a FTT [17]. Our study was not designed to propose cut-off values of postprandial TAG response and more research with the use of Lipotest^®^ is necessary.

If the results of this study apply to other populations with increased CVD risk, a FTT using Lipotest^®^ can be performed once after 8 h fasting and plasma/serum TAG concentrations can be measured at 4 h postprandially in order to capture postprandial response. We found that the incremental AUC of plasma TAG is not affected by BMI and, therefore, the Lipotest^®^ can be used irrespective of the BMI status. This approach may help in the identification of subjects with exaggerated postprandial lipemia and can motivate healthcare professionals to interventions aiming at reduction of residual CVD risk. While this clinical application of the FTT using Lipotest^®^ requires only the fasting and 4 h TAG measurements, the increasing use of multisampling FTT protocols in research studies may led to interest in the additional information that potentially may be derived from the lipid patterns and lipid sub-fractions that arise during the test.

The study is not without limitations. First, it was performed in relatively young healthy men; therefore, the results cannot be extrapolated to other populations. Second, obese subjects usually have pronounced increase in postprandial TAG after consumption of a fat-rich meal. In this study, obesity was not an exclusion criterion but only five subjects were obese (BMI values were between 30 and 34 kg/m^2^) and separate analysis in the obese participants was not possible. Third, larger studies involving lean and obese subjects of both gender, women of reproductive age according to their menstrual phase of the cycle (luteal or follicular phase), and subjects with comorbidities as well as patients with diabetes could provide more information on the reliability and reproducibility of the results. Four, more research is needed to study whether Lipotest^®^ testing has advantages over non-fasting TAG determination, as recently proposed [52], for CVD risk stratification.

In conclusion, this study has shown that a FTT using Lipotest^®^ as a standardized meal has high precision and good reproducibility for the study of postprandial TAG response in healthy individuals. A single determination of plasma TAG concentration at 4 h after Lipotest^®^ consumption captures peak postprandial TAG response. More research is necessary to examine the validity of the test in other populations and its potential advantages over non-fasting TAG determination.

## Abbreviations

CVD: Cardiovascular disease
FFT: Fat tolerance test
TAG: Triacylglycerol
OGTT: Oral glucose tolerance test
CV: Coefficient of variation
ICC: Intraclass correlation coefficient
AUC: Αrea under the curve
iAUC: Incremental area under the curve
iC_max_.: Maximal concentrations
*T*_max_: Time to maximal concentrations
BMI: Body mass index
HDL-C: High density lipoprotein cholesterol
LDL-C: Low density lipoprotein cholesterol
CM: Chylomicrons
VLDL: Very low-density lipoproteins
