# Erratum to: Assessment of the Validity and Reproducibility of a Novel Standardized Test Meal for the Study of Postprandial Triacylglycerol Concentrations

**DOI:** 10.1007/s11745-017-4284-8

**Published:** 2017-08-29

**Authors:** Nikolaos Tentolouris, Panagiotis T. Kanellos, Evangelia Siami, Elpida Athanasopoulou, Nikolaos Chaviaras, Genovefa Kolovou, Petros P. Sfikakis, Nikolaos Katsilambros

**Affiliations:** 1 First Department of Propaedeutic Internal Medicine, Medical School, Laiko General Hospital, National and Kapodistrian University of Athens, 17 Agiou Thoma Street, 11527 Athens, Greece; 2 Cardiology Department, Onassis Cardiac Surgery Center, Athens, Greece

## Erratum to: Lipids (2017) 52:675–686 DOI 10.1007/s11745-017-4275-9

The article “Assessment of the Validity and Reproducibility of a Novel Standardized Test Meal for the Study of Postprandial Triacylglycerol Concentrations”, written by Nikolaos Tentolouris, Panagiotis T. Kanellos, Evangelia Siami, Elpida Athanasopoulou, Nikolaos Chaviaras, Genovefa Kolovou, Petros P. Sfikakis, Nikolaos Katsilambros, was originally published electronically on the publisher’s internet portal (currently SpringerLink) on 26 June 2017 without open access. The original article was corrected.

With the author(s)’ decision to opt for Open Choice the copyright of the article changed on 31 August 2017 to © The Author(s) 2017 and the article is forthwith distributed under the terms of the Creative Commons Attribution 4.0 International License (http://creativecommons.org/licenses/by/4.0/), which permits use, duplication, adaptation, distribution and reproduction in any medium or format, as long as you give appropriate credit to the original author(s) and the source, provide a link to the Creative Commons license, and indicate if changes were made.

